# General Pathology

**Published:** 1988-08

**Authors:** J. D. Davies


### Book Review

### GENERAL PATHOLOGY

**J. B. Walter and M. S. Israel**
**6th edition, 739+×pages, 1988**
**Churchill-Livingstone, Edinburgh, price: £49.50**
**ISBN 0 443 03089 8**

Some books are immensely influential. Walter and Israel's 'General Pathology' has proved to be one of this kind.

The first edition appeared in 1963, probably before most readers of this Journal had even qualified. This—the sixth edition—was published late last year. How has it fared in the last quarter century?

The main problem in the field of general pathology texts is that there is none which is entirely satisfactory. This has been a problem for teachers at undergraduate and postgraduate levels for a long time. Short paperback books are numerous, and now probably the best is that of Woolf. However they are not expansive enough for the keener student, and may be insufficient for all postgraduate purposes. The American texts, though overcoming some of these shortcomings are formidably verbose and expensive—or were the latter until currency exchange rates began to change.

Walter and Israel still covers most of the field. Armed with the information in this book no postgraduate candidate could possibly fail any Primary FRCS examination. Nor could any student facing the perils of 2nd MB fail to pass with ease.

The text is clear, and most of the illustrations are helpful. What are the reservations? For me, it is merely a question of ageing. Most of the book has changed little over the years. In this latest edition the immunology and genetics have received the welcome attentions of two new collaborators. None the less, this is a running repair job. A completely new British and full general pathology book is urgently required. I don't mean a 'pathobiology' text, but a solidly medical textbook dealing with the general aspects of disease processes faced in the clinical context. Best done—like Walter and Israel—by very few authors with width of experience, but this time with the 1990's in view, instead of the later 1960's. Essentially the topics chose themselves, and their order of presentation needs review in the new circumstances. However I will finish by saying that if anyone does write such a book—and it lasts for as long as Walter and Israel—then they really do deserve the praise which Walter and Israel have certainly merited in the last 25 years.

J. D. DAVIES

